# Epicardial Pacemaker Lead Related Cardiac Strangulation: The Importance of Early Recognition

**DOI:** 10.1007/s00246-025-03792-x

**Published:** 2025-01-29

**Authors:** Scott Kendall, Rihab Agouba, Jane Murray, Margaret Louise Morrison, Brian McCrossan, Brian Grant, Andrew Sands, Frank Casey, Lars Nolke

**Affiliations:** 1 Paediatric Cardiology Department, Royal Belfast Hospital for Sick Children, Belfast, UK; 2 Queens University Belfast, University Road, Belfast, UK; 3 Cardiothoracic Department, Children’s Health Ireland at Crumlin, Dublin, Ireland

**Keywords:** Pacemaker, Congenital heart disesae, Complication

## Abstract

Lead strangulation is a dangerous complication of epicardial pacemaker insertion. This complication has been increasingly highlighted lately. Our institution has recently identified four cases over the past five years. This study’s aim was to 1) identify risk factors for strangulation and 2) prospectively screen existing epicardial pacemaker patients for unrecognized strangulation or features that would prompt closer review. Patients known to the pacemaker clinic with epicardial pacemakers inserted from 2005 to 2023 were included. Electronic health records were used to locate all subjects and gather data. Risk factors were identified using Firth’s penalized method of logistic regression. Forty-five patients were included, of which four (8.8%) had evidence of strangulation. Posterior–anterior (PA) chest radiographs all demonstrated characteristic looping patterns of the pacing leads, with confirmation on CT angiography. All affected patients underwent revision surgery. Implantation at a weight of less than 6.5 kg was associated with a significantly increased incidence of strangulation (OR 25, *P* 0.044). Other factors including lead length, presence of structural cardiac disease, and dual-chamber insertion were not statistically significant. No patients who were prospectively screened were found to have strangulation. Children undergoing insertion of a pacemaker early in infancy are at particularly high risk of strangulation and should be closely monitored following surgery. Regular chest radiography (every three years) to screen for this complication is advised. Larger multi-center studies to pool data for this relatively rare complication may help identify other risk factors for strangulation.

## Introduction

Cardiac strangulation is a serious complication of epicardial pacemaker insertion necessitating surgical revision. Although traditionally thought to be infrequent, the reported incidence has been increasing [1, 2].

At our institution, four cases of strangulation were identified in the past five years. We therefore decided to screen all pediatric epicardial pacemaker patients. This study aimed to 1) identify risk factors for strangulation and 2) prospectively screen existing epicardial pacemaker patients for unrecognized strangulation or features that would prompt closer review.

## Methods

Children attending the local pacemaker clinic who had an epicardial pacemaker (including non-functional devices) were included. Data collected included indication for pacemaker, age at the time of insertion, modality of the pacemaker, weight at the time of insertion, sex, pacemaker lead length, revision surgeries, and time since insertion. Two pediatric cardiology consultants reviewed PA chest radiograph (PA CXR) of all children for evidence of potential strangulation, and those who had not had a recent study in the past three years were flagged for repeat imaging.

Statistical analysis was performed using SPSS (Version 29, IBM, New York). Continuous variables were reported as medians (due to the non-parametric data). Potential risk factors for strangulation were analyzed using Firth’s penalized method of logistic regression, and the 2-sided *P* value of < 0.05 was considered statistically significant.

## Results

Forty-five patients were identified who attend the pediatric pacemaker clinic and have an epicardial pacemaker or redundant epicardial leads in situ. There were 21 girls and 24 boys who underwent pacemaker implantation at a median age of 13 months (Interquartile Range IQR 3.9, 31.5), and the median weight was 9.8 kg (IQR 5, 15). Four cases (8.8%) of strangulation were identified. 28 patients (62%) had a single-chamber pacemaker inserted, while 17 patients (38%) had a dual-chamber system inserted. 36 patients (80%) had undergone follow-up for four or more years with the remaining nine undergoing follow-up for three years or less. The lead length varied from 25 to 69 cm. 29 patients (64.4%) had congenital heart disease. Indications for pacemaker insertion included high-grade atrioventricular block (congenital and post-operative), sinoatrial node disease, complex congenital heart disease (e.g., congenitally corrected transposition of the great arteries/isomerism of the left atrial appendages) (Table 1). The median follow-up period was four years.

**Table 1:** Patient characteristics

	N (%)*
Male/Female	24 M/21 F (54/46)
Age at Insertion (months) Median	Median 13 (range: 0–136)
Weight at insertion (kg)	Median 9.8 (range 2.1–25)
Weight < 6.5 kg	21 (46.7)
Lead Length (cm)	Median 35 (Range 25–69)
Dual Chamber System Inserted	28 (62)
Length of Follow Up ≥ 4 years	36 (80)
Structural Heart Disease	29 (64)

^*^Unless otherwise noted, *cm* centimeters

Four patients had evidence of strangulation, all with a background of congenital complete heart block, with otherwise normal cardiac anatomy, who had an epicardial pacemaker inserted either in the neonatal period or in one case at 4 months of age (Table 2). All of these cases were identified prior to the review of the remaining pacemaker patients.

**Table 2:** Strangulation patient characteristics

Case	Diagnosis	Age at Implantation (Months)	Weight at implantation (Kg)	Dual Chamber (Y/N)	Age at diagnosis of Strangulation (Months)	Lead length (cm)
1	CCHB	0	2.3	N	106	35
2	CCHB	4	6.5	Y	96	35
3	CCHB	0	3.3	N	145	35
4	CCHB	0.5	3.2	N	153	35

*CCHB* Congenital Complete Heart Block

**Fig. 1:**
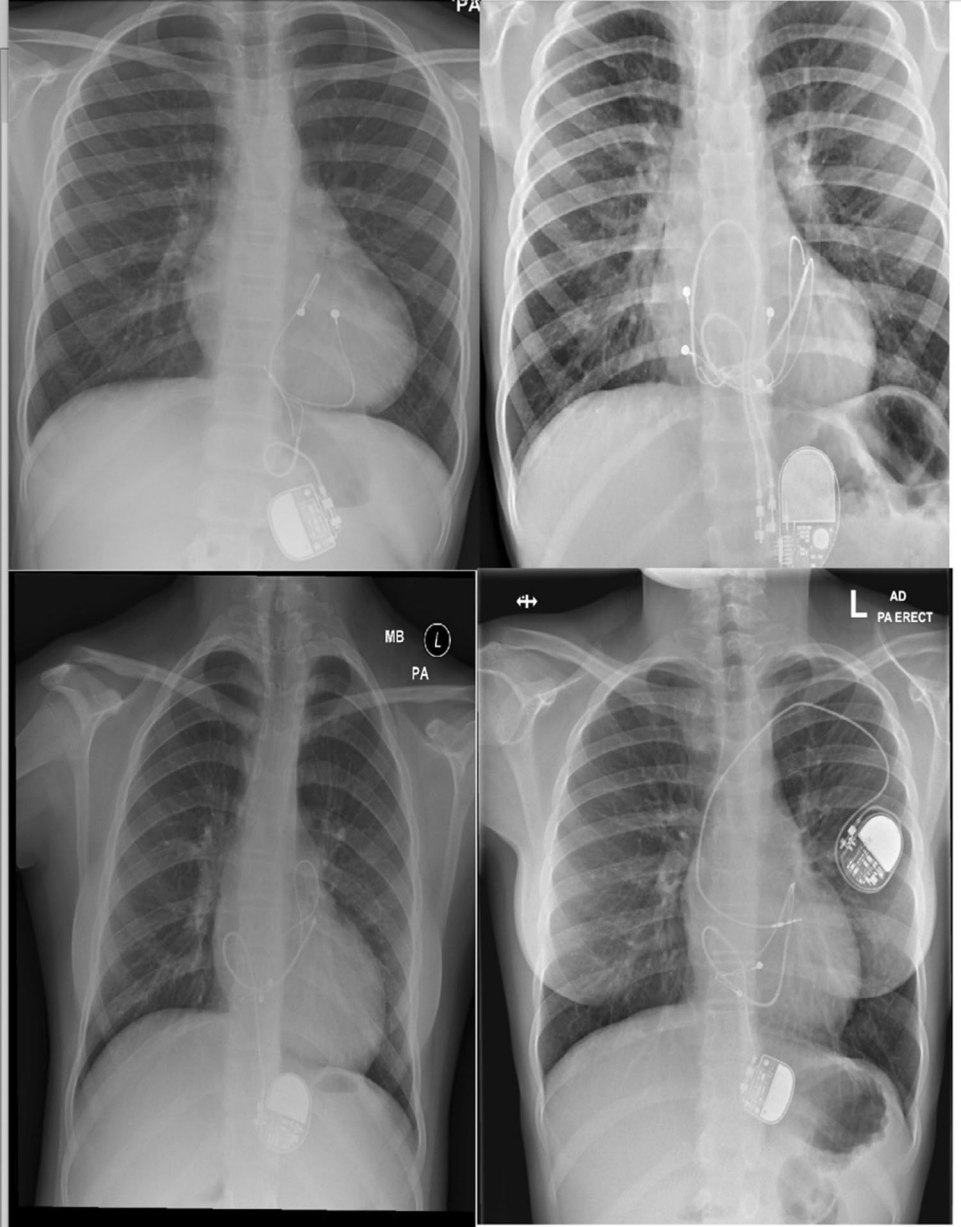
PA CXRs of all children diagnosed in our institution with cardiac strangulation, evidence of looping of leads seen in all four cases

**Fig. 2:**
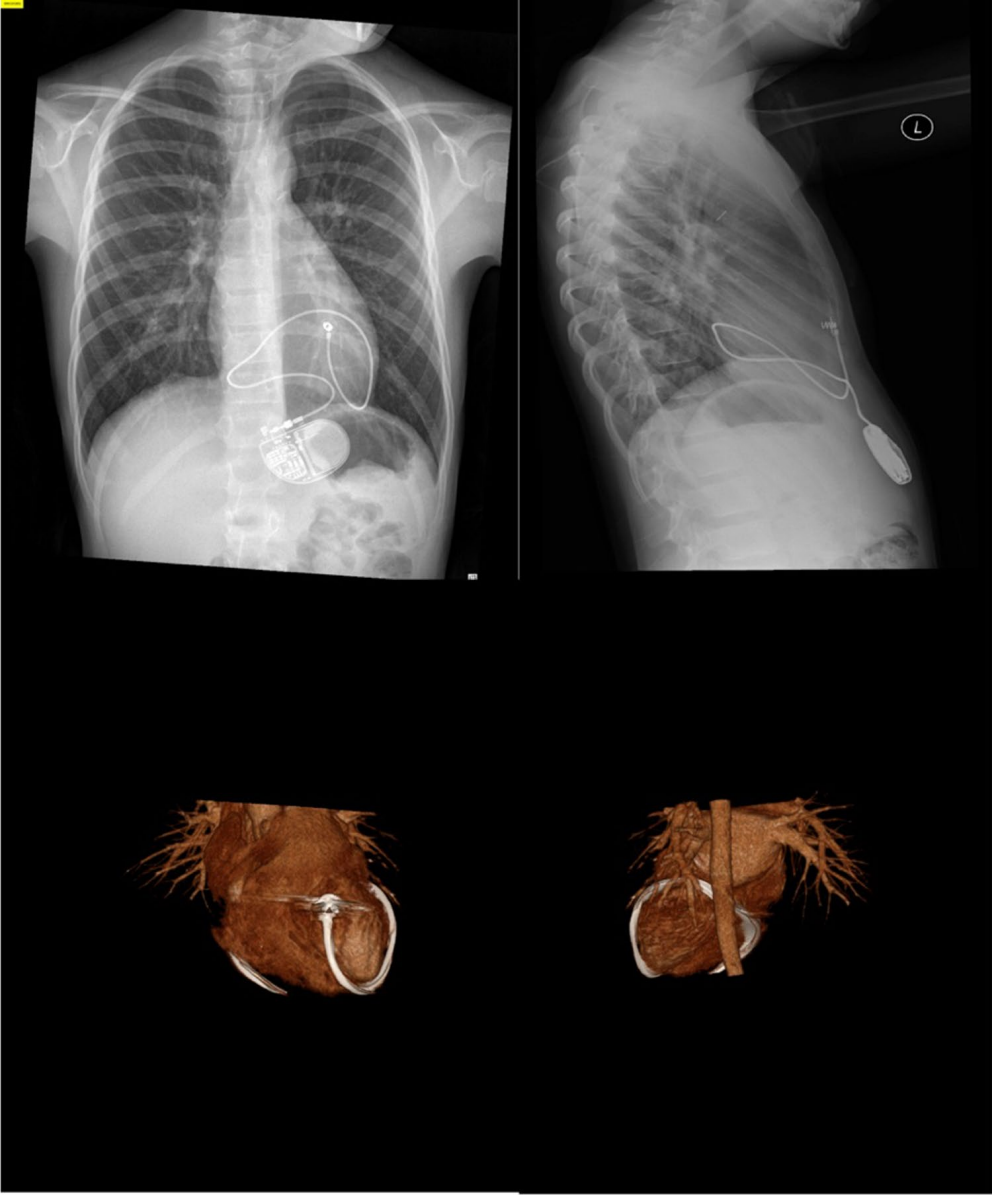
Appearance of a case initially mimicking strangulation with the appearance of plain radiography appearance (panel 1 &2), CT angiography confirming no evidence of indentation on the heart (panel 3&4)

**Fig. 3:**
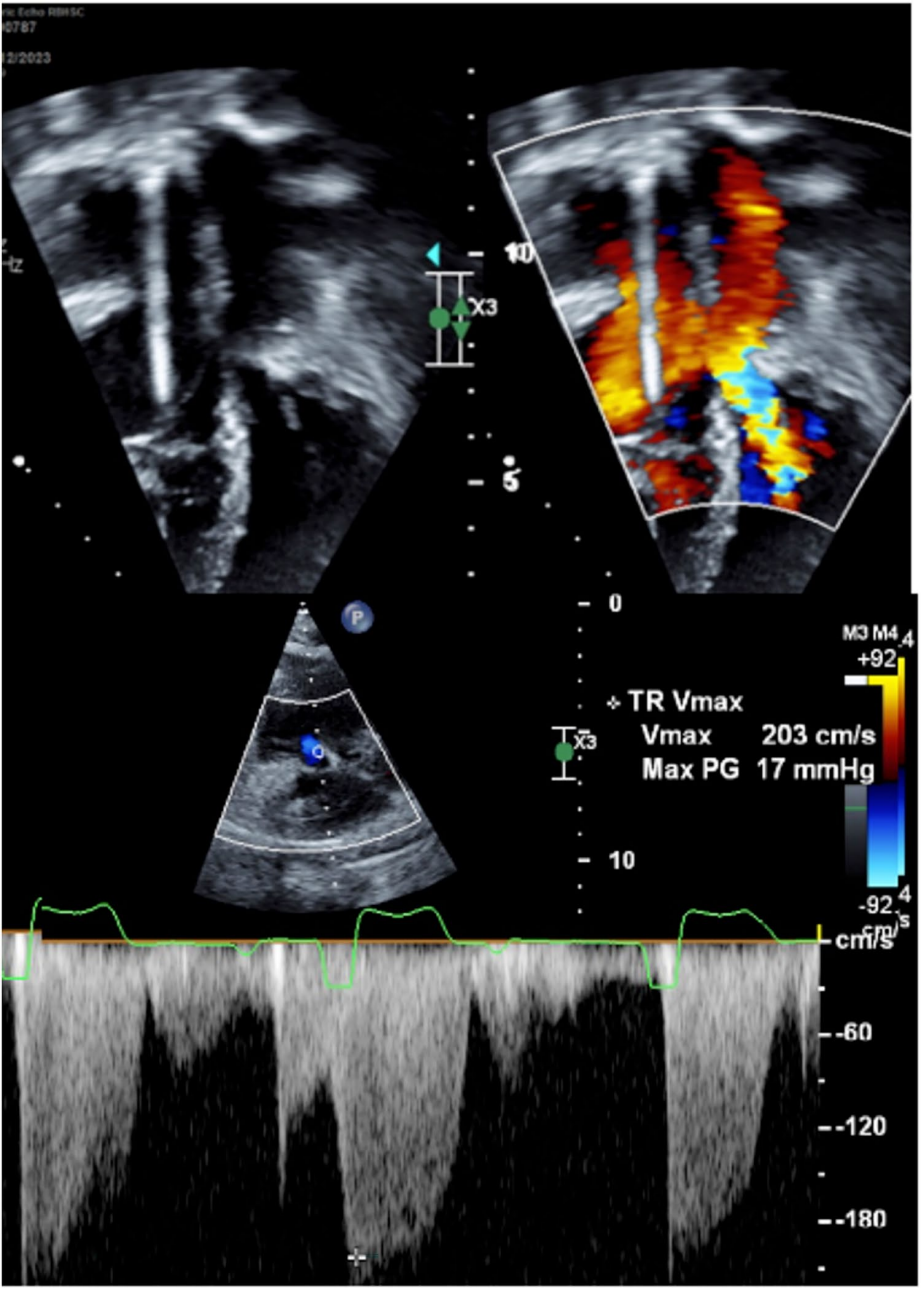
Echo findings of one patient demonstrating the epicardial lead crossing the Tricuspid valve, resulting in tricuspid regurgitation. There is also mitral valve inflow acceleration

Children with strangulation had consistent features on PA CXR including excess lead length and leads encircling the heart in a looping fashion (Fig. 1). These features were therefore screened for in all children with epicardial pacemakers. If there were concerns for strangulation, a lateral CXR was obtained and if concerns remained a CT angiogram was performed. One case had concerning features suggestive of looped leads, but a CT showed not indentation on the heart, with no evidence of impingement of coronary vessels or AV Valves. (Fig. 2). Echocardiographic features suggestive of strangulation included mitral valve acceleration in three of the four case and tricuspid incompetence with evidence of the lead distorting the tricuspid valve in one case (Fig. 3).

Potential risk factors analyzed using Firth’s Logistic regression included weight less than 6.5 kg, lead length, insertion of a dual pacemaker, and structural heart disease. Weight < 6.5 kg was the only feature significantly associated with strangulation, with an odds ratio = 25.7 CI (2.07–10938) *P* = 0.044. (Table 3).

**Table 3:** Potential Risk Factor analysis for cardiac strangulation

	Odds ratio	95% CIs	***P*** Value
< 6.5 kg	25.7	2.07–10938	**0.04**
Increasing Lead Length	1.47	0.33–6.6	0.57
Dual Chamber Insertion	0.66	0.036–5.8	0.72
Structural Heart Disease	0.11	0.01–1.05	0.06

*Cis* Confidence intervals

## Discussion

The reported incidence of cardiac strangulation is increasing [1.2]. This is likely due to increased recognition and surveillance. One recent study demonstrated that 5% of patients with epicardial leads had evidence of coronary compression [1]. Strangulation caused by leads looping around the heart may impair atrioventricular valve (AV), coronary artery, or great artery function resulting in heart failure, ischemia, or arrhythmia [3]. Symptoms of strangulation vary or may be absent [2]. When present, symptoms include fatigue, breathlessness, syncope, and chest pain [2, 4, 5]. Increased cardiac demand such as exercise or anesthesia has been associated with unmasking symptoms [5]. Signs can include edema or a new murmur heralding AV valve regurgitation [2, 5].

Given the insidious nature of the condition, it is important to screen for strangulation regularly in all children with epicardial pacemakers. However, very few studies have identified risk factors for occurrence and low study participant numbers hamper those that have. One study demonstrated no individually significant risk factors but the combination of < 6 month at insertion, weight < 7.3 kg, dual-chamber pacemaker, absence of post-pericardiotomy syndrome, and absence of post-operative infection had an odds ratio = 17 of strangulation in their population [4]. Another case series of cardiac strangulation events also noted that it was seen more often in children having epicardial pacemakers inserted at a younger age (neonate to 3-year olds) [3]. This was echoed in another case series which found that 16/22 cases involved leads implanted in the first year of life with 10/22 being implanted in the neonatal period [2]. In contrast, coronary compression was not associated with lead type, age, or weight in another study [1].

Weight less than 6.5 kg was associated with an increased odds ratio = 25 for strangulation in our population (*P* = 0.04). Other factors including dual-chamber pacemakers and greater lead length were not found to be significantly associated with strangulation. Age at insertion was purposefully not included in statistical analysis as weight was felt to be a more important variable. The weight of the child rather than their age is a more helpful, precise factor given the variation in weight at the same age, especially when comparing children with structural heart disease to those without. The four cases described in this publication are strikingly similar with regard to indication weight and age of diagnosis of strangulation.

Various surgical methods have been described with the aim of avoiding strangulation, these include placement of excess lead on the diaphragmatic surface of the pericardium, the pleural space [6], or on the anterior surface of the myocardium [4]. Efforts should be made to minimize redundant lead within the pericardial sac while also allowing for linear growth [4]. Use of a Teflon sheet to separate the leads from the heart or the use of a small counterclockwise loop are other suggested safer techniques [7]. Intraoperatively, attention should be paid to the course of the coronary arteries and leads deliberately routed away from them, bearing in mind the possibility of leads being wedged anteriorly between the sternum and the heart [3].

Previous studies have provided recommendations to regularly perform PA and lateral CXR which are reported to have a sensitivity of 57% and a specificity of 96% for strangulation [1]. In the case of suspected strangulation, CT angiography can provide a definitive diagnosis and aid surgical planning for lead replacement. Three yearly CXRs have been suggested as a reasonable interval for children with epicardial lead placement [3]. It is important to remember that children with redundant leads can suffer strangulation, as was the case with our fourth patient (Fig. 2). A recent case report of the first adult death due to cardiac strangulation [8] should perhaps prompt ongoing review even after linear growth is completed. Our institution has opted to perform three yearly PA CXR and only perform lateral CXRs if the findings are suspicious to limit radiation exposure.

This study has several limitations, including it is retrospective in nature and includes small numbers of patients from a single, relatively small institution. It also was conducted over a period during which surgical technique has changed. Pooling results with other institutions would aid further analysis and increase precision of risk factors.

On balance given this study’s findings in combination with previous published works, we feel it is fair to conclude that strangulation is more common than has been reported and that smaller size at insertion of leads is a risk factor. Regular surveillance, for example, every three years, through plain radiography is recommended to prevent further preventable deaths.

## Data Availability

SK can provide the data on reasonable request.
